# Females and Males Rely on Different Cortical Regions in Raven’s Matrices Reasoning Capacity: Evidence from a Voxel-Based Morphometry Study

**DOI:** 10.1371/journal.pone.0093104

**Published:** 2014-03-25

**Authors:** Wenjing Yang, Peiduo Liu, Dongtao Wei, Wenfu Li, Glenn Hitchman, Xueping Li, Jiang Qiu, Qinglin Zhang

**Affiliations:** 1 Key Laboratory of Cognition and Personality (SWU), Ministry of Education, Chongqing, China; 2 Faculty of Psychology, Southwest University, Chongqing, China; 3 Institute of Education, China West Normal University, Nanchong, China; University Children’s Hospital Tuebingen, Germany

## Abstract

Raven’s Matrices test (RMT) is a non-verbal test designed to assess individuals’ ability to reason and solve new problems without relying extensively on declarative knowledge derived from schooling or previous experience. Despite a large number of behavioral studies that demonstrated gender differences in Raven’s Matrices reasoning ability, no neural evidence supported this difference. In this study, voxel-based morphometry (VBM) was used in an attempt to uncover the gender-specific neural basis of Raven’s Matrices reasoning ability as measured by the combined Raven^’^s Matrices test (CRT) in 370 healthy young adults. The behavioral results showed no difference between males and females. However, the VBM results showed that the relationship between reasoning ability and regional gray matter volume (rGMV) differed between sexes. The association between CRT scores and rGMV in the dorsolateral prefrontal cortex (associated with visuospatial ability) was significantly greater in males than in females, whereas the reverse was true for the inferior frontal cortex (relating to verbal reasoning ability) and the medial frontal cortex (engaged in information binding) where the association was greater in females. These findings suggest that males and females use differently structured brains in different ways to achieve similar levels of overall Raven’s Matrices reasoning ability.

## Introduction

Raven’s Matrices Test (RMT) is a non-verbal test designed to assess individuals’ ability to reason and solve new problems without relying extensively on declarative knowledge derived from schooling or previous experience [1]. These matrices are composed of a series of nonverbal pictures, each with a missing element that completes a pattern. Subjects are asked to identify the missing element that completes the pattern. Good RMT performance requires that subjects perceive the relationships between cells in the matrices, determine the relationships between the columns and rows of the matrices and then integrate this information. The linguistically minimized nature of the RMT test allows for the measurement of reasoning capacity without the influence of language, education, and cultural factors. Thus, the RMT is considered one of the best indexes of individual differences in reasoning ability [2].

Sex differences in Raven’s Matrices reasoning ability are among the most controversial and interesting topics in this area of study. The results of studies to date have not allowed researchers to reach a clear consensus regarding their conclusions. A number of researchers contest that no gender differences exist in Raven’s Matrices ability [3]–[5]. In particular, Lynn et al. (2004) reported that no statistically significant difference existed between scores obtained by boys and girls on the Standard Progressive Matrices test for a sample of nine-hundred and twenty 7–10 year olds in Mexico. Furthermore, a recent standardization of the Progressive Matrices in Syria for people aged 7 to 18 years also found no sex differences [3]. However, some investigations of sex differences in the Raven’s Matrices test showed a male advantage [6]–[9]. Lynn (1998) has analyzed data from England, Hawaii, and Belgium and found that males outperformed females in the Standard Progressive Matrices Test. This result was repeated in a study using the Advanced Progressive Matrices [8]. Meanwhile, some other studies have observed an advantage for females [3], [10]. For example, Khaleefa and Lynn (2008) reported on a large standardization sample of 6–11 year olds who were tested using the Colored Progressive Matrices test in the United Arab Emirates. Girls performed significantly better than boys, but the difference was only small. In another study, the Standard Progressive Matrices was standardized on a sample of 6529 8–15-year olds in Kuwait. The results showed that a small sex difference favored girls [10].

A large body of functional magnetic resonance imaging (fMRI) and positron emission tomography (PET) studies have tried to investigate the neural mechanisms of Raven’s Matrices test [11]–[18]. Some PET studies have noted strong activation in the left parieto-occipital region during Raven’s test performance [19], [20]. Prabhakaran et al. (1997) examined brain activation during solving the Raven’s Progressive Matrices problems in seven young healthy participants. Right frontal and bilateral parietal regions of the brain were activated more by the visuospatial reasoning required by Raven’s test problems compared with control problems. Another study independently manipulated relational complexity and distractor demands in a RMT-like task and observed that bilateral DLPFC activation, extending into FPC in the left hemisphere, increased with relational complexity [15]. Generally, results from fMRI and PET studies suggest that Raven’s Matrices reasoning is associated with activation of a network of frontal and parietal brain regions, specifically the dorsolateral prefrontal cortex (DLPFC), the superior parietal lobule and intraparietal cortices [21].

To the best of our knowledge, no study has explored the gender-specific neural mechanism of Raven’s Matrices reasoning ability. Some studies identified two general problem solving strategies that could be used to solve the items of the Raven’s Matrices. One is the visual strategy, which involves applying operations of visual perception, such as the superimposition of images upon each other. The other is the verbal strategy, which consists of applying logical operations to features contained within the problem elements [22], [23]. Previous studies have already demonstrated that males and females may use different approaches to solve Raven’s Matrices spatial problems [24], [25]. It is well known that different brain regions underpin visual and verbal abilities [26]–[35]. If males and females use different strategies to solve Raven’s reasoning problems, it is reasonable to hypothesize that there will be gender-specific neural correlates of Raven’s Matrices reasoning ability [36]. Because functional imaging can only measure active processing which is constrained by the task, it can’t identify the neural substrates of sex-specific abilities. Structural imaging studies are particularly useful for investigating the anatomical correlates of personal characteristics involving a wide range of behaviors [37]. In this study, voxel-based morphometry (VBM) was used to explore the gender-specific neural correlates of Raven’s Matrices reasoning ability. Haier et al. [38] used the Wechsler Adult Intelligence Scale (WAIS) to examine sex differences related to general intelligence. The results showed that the correlation between gray matter volume and general intelligence was stronger in the frontal and parietal lobes for males, whereas a stronger correlation was found in the frontal lobe along with Broca’s area for females. It is well known that RMT has been widely accepted as measurement of general intelligence [2], [39]–[42]. Based on the previous researches, we hypothesized that males with higher reasoning scores may have an increased regional gray matter volume (rGMV) in the parietal or frontal regions associated with visuospatial ability, whereas females with higher reasoning scores may have a larger rGMV in the inferior frontal regions associated with verbal-related abilities.

## Materials and Methods

### Ethics Statement

The experiment was approved by the Academic Committee of the School of Psychology and the Brain Imaging Center Institutional Review Board of Southwest University in China. All participants signed an informed consent form prior to their inclusion in the study.

### Participants

A total of 384 right-handed, healthy volunteers (194 females; mean age: 19.82±1.31; and 190 males; mean age: 20.22±1.37) from the Southwest University in China participated in this study, as part of our ongoing project to examine the association among brain imaging, creativity, and mental health. All participants were native Chinese speakers, and had normal or corrected-to-normal vision. Participants were screened to confirm healthy development by a self-report questionnaire survey before the scanning, and thus, those participants who had a history of psychiatric or neurological disorders, received mental health treatment or taken psychiatric medications were excluded. Among the participants, nine were excluded because they did not take part in the behavioral portion of the study. Another five participants were removed because of excessive head motions. Therefore, 370 participants (190 females, 180 males) were included in the VBM analyses.

### Behavioral Examination

Raven’s reasoning matrices are available in three different forms for different aged participants: the Standard Progressive Matrices, the Colored Progressive Matrices and the Advanced Progressive Matrices [43]. In this study, we chose to use the Chinese version of the RMT, the combined Raven’s matrices test (CRT) [44]–[46]. Participants had up to 40 minutes to finish this test. The CRT consists of the Raven’s standard progressive Matrices (C, D and E sets) and Raven’s colored progressive Matrices (A, B and AB sets). Given that the Colored Progressive Matrices were designed for children aged 5 to 11 years old, some researchers combined the colored and standard Matrices to make the test generalizable to more people. The CRT can be applied to people aged 5 to 75.

### Image Acquisition

MR images were acquired on a 3.0-T Siemens Trio MRI scanner (Siemens Medical, Erlangen, Germany). High-resolution T1-weighted anatomical images were acquired using a magnetization-prepared rapid gradient echo sequence [repetition time (TR) = 1900 ms; echo time (TE) = 2.52 ms; inversion time (TI) = 900 ms; flip angle = 9 degrees; resolution matrix = 256×256; slices = 176; thickness = 1.0 mm; voxel size = 1×1×1 mm].

### Preprocessing of Structural Data

The MR images were processed using SPM8 (Wellcome Department of Cognitive Neurology, London, UK; www.fil.ion.ucl.ac.uk/spm/) implemented in Matlab 7.8 (MathWorks Inc., Natick, MA, USA). Each MR image was first displayed in SPM8 to screen for artifacts or gross anatomical abnormalities. For better registration, the reorientation of the images was manually set to the anterior commissure. The images were segmented into gray matter (GM), white matter (WM), and cerebrospinal fluid by using the new segmentation in SPM8. Subsequently, we performed Diffeomorphic Anatomical Registration through Exponentiated Lie (DARTEL) algebra in SPM8 for registration, normalization, and modulation [47]. To ensure that regional differences in the absolute amount of GM were conserved, the image intensity of each voxel was modulated by the Jacobian determinants. Then, registered images were transformed to Montreal Neurological Institute (MNI) space. Finally, the normalized modulated images (GM and WM images) were smoothed with a 10 mm full-width at half-maximum Gaussian kernel to increase signal-to-noise ratio.

### Statistical Analysis

Statistical analyses of GMV data were performed using SPM8. This study aimed to investigate whether the relationship between rGMV and Raven’s reasoning scores differed between males and females. Sex differences were tested using the condition by covariate interaction analysis [37], [48]. In the whole brain analysis, sex was treated as a condition. To control for possible confounding variables, age, scores of the CRT, and global volumes of GM were entered as covariates into the model. Aside from total brain volume, all covariates were modeled to make the unique relationship of each covariate with rGMV evident for each sex. The interaction effects between sex and the Raven’s reasoning score on the rGMV was assessed using t-contrasts.

To avoid edge effects around the borders between GM and WM, an absolute threshold masking of 0.2 was used, meaning that voxels with gray matter values lower than 0.2 were excluded from the analyses. For all analyses, the cluster-level statistical threshold was set at P<0.05, and corrected at the non-stationary cluster correction [49] with an underlying voxel level of P<0.001. This was an exploratory study, therefore, we did not use the FDR or FEW approaches for multiple comparison correction.

## Results

### Sample Descriptive Statistics

A total of 370 healthy participants (190 females, 180 males) were included in the VBM analysis. The mean CRT scores were (66.13±3.13) for males and (66.40±3.05) for females. No significant gender difference (*P*>0.05) in CRT scores was found between females and males.

### VBM Results

**Figure 1:**
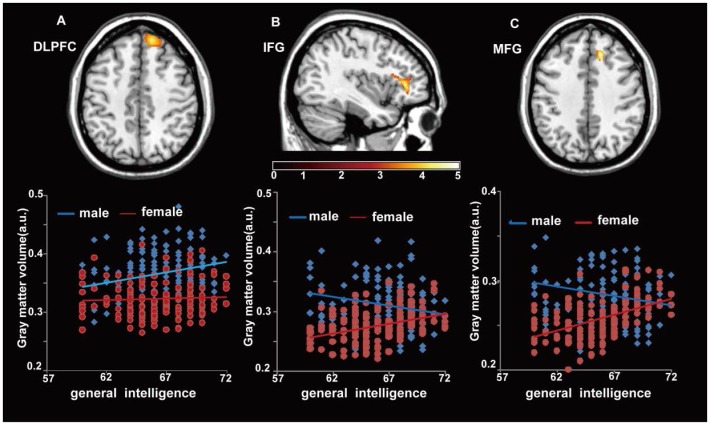
Sex modulates the effect of reasoning ability on gray matter in the Dorsolateral prefrontal cortex (DLPFC), the Medial frontal cortex (MFC), and the Inferior frontal cortex (IFC). The color density represents the T score. The scatter plot between CRT scores and the rGMV is shown for illustration purpose only.

A voxel-wise ANCOVA analysis showed that there was an interaction effect between sex and CRT scores on GMV in the following three regions: the first region was in and adjacent to the right dorsolateral prefrontal cortex (DLPFC, BA9, cluster size = 1130, *t = *4.27), the second region was spread around the left IFC (BA45,cluster size = 1106, *t = *5.21), and the third region was around the right medial frontal cortex (Medial FC, BA32, cluster size = 2018, *t = *4.82) (see Table 1). The CRT scores were positively correlated with GMV in the right DLPFC (r = 0.217, *p = *0.006) for males, whereas no significant correlation was found for the females in this region (r = 0.02, *p = *0.776) (Figure 1A). The CRT scores of females were positively correlated with the GMV in one cluster adjacent to the left IFC (r = 0.176, *p = *0.011), whereas a negative correlation was found for males (r = −0.284, *p = *0.000) (Figure 1B). In addition, the Raven test scores for females also showed a positive correlation with the GMV in the cluster adjacent to the right Medial FC (r = 0.184, *p = *0.008), whereas the scores for males were negatively correlated with the GMV of this cluster (r = −0.281, *p = *0.000) (Figure1C). The scatterplot between CRT scores and regional gray matter volume (rGMV) is shown for illustration purpose. They likely overestimate the effects because the signal of peak voxel was extracted.

**Table 1:** Sex modulates the effect of reasoning ability on gray matter in the following regions.

	Brain region	BA	MNI coordinate			Cluster size	Peak *t* value
			X	Y	Z		
Males>female	DLPFC	9	13	46	45	1130	4.27
Female>male	MFC/SMFC	32	15	31	39	2018	4.82
	IFC	45	−39	34	5	1106	5.11

Note: BA = Broadman areas; DLPFC = Dorsolateral prefrontal cortex; MFC = Medial frontal cortex; SMFC = Superior medial frontal cortex; IFC = Inferior frontal cortex.

## Discussion

This study investigated the sex-specific neuroanatomical differences underlying the reasoning ability as measured by the CRT in a large sample of participants. VBM analysis revealed distinct differences in the contribution of various cortical regions to reasoning ability between males and females. Males’ reasoning scores were positively correlated with the rGMV in the right DLPFC, whereas females’ scores showed a positive correlation with the rGMV in the left IFC and right Medial FC. Our results may indicate that females and males appear to rely on different neural substrates to achieve comparable reasoning performances in the CRT. Some studies have suggested that Raven’s reasoning Matrices may measure different abilities in males and females [24]. Our study provided a basis for explaining these findings in terms of neurological structure substrates.

The positive correlation between the rGMV of the DLPFC and the reasoning scores for the male participants indicates that males may rely more on the DLPFC to obtain higher reasoning scores [50]. DLPFC has been documented to serve an important function in maintaining spatial information and using this information to guide a correct response [26], [27], [51]–[54]. fMRI studies have revealed significant activation in the lateral prefrontal cortex while subjects performed visuospatial tasks such as the spatial navigation task, the delayed-response task, the mental rotation task or some tasks that require the temporary maintenance of spatial information [26], [52], [55]–[57]. Some evidence from visuospatial tasks which requires spatial representation and memory, such as the mental rotation task and the spatial navigation task, suggest that males have an advantage in visuospatial ability [28], [58]–[60]. Other studies have demonstrated that visual strategies are important in solving Raven’s matrices [22], [23] and males tend to use their visual-spatial ability while solving these items [8], [22], [61]. Males’ habitual use of this ability may cause an augmentation of the brain regions which underpin it [36]. Our results may therefore indicate that males rely more than females on the DLPFC associated with the visuospatial ability to solve the Raven’s Matrices reasoning problems.

The increased rGMV in the IFC for females may suggest that females obtain higher reasoning scores as measured by the CRT through verbal-analytic reasoning ability. A large body of neuroimaging evidence has shown that the left IFC is engaged in verbal correlated processes, such as phonologic and semantic operations [62]–[65]. The mental logic theory of reasoning suggests that reasoning relies on a language-like structure and should be supported by the language areas [66], [67]. Previous studies showed that verbal-analytic reasoning was an important strategy in solving spatial-format problems and females tend to use their verbal ability when solving these reasoning problems [23], [68]. This strategy requires the existence of a verbal representation of the stimuli and applies logical operations to features contained within elements of the problem matrices [22], [61]. Females’ frequent use of their verbal ability may be the underlying cause of the positive correlation between the reasoning ability and the rGMV of IFG, which is thought to underpin this ability [36]. The increased rGMV in the IFC also suggests that females rely more than males on the IFC when solving spatial-format problems.

In addition, the results also showed that an increased rGMV for females in the Medial FC was associated with a higher reasoning score. Some studies showed that some answers to simple items in the Raven’s Progressive Matrices tests are easily obtained by perception of the pattern as a gestalt, such that the appropriate piece for its completion can be identified without the use of reasoning [69]. During this matching process, it is thought that participants retain the original pattern in working memory and try to judge whether the optional pattern can match the original one. The initial pattern must be kept online, and these two discontinuous events must be bound together for a conclusion to be made. Some neuroimaging studies have shown that the Medial FC is engaged in bounding items together to obtain a successful associative memory of information [70]–[72]. Thus, the positive correlation for females between the rGMV of the Medial FC and reasoning ability may suggest that females rely more on the ability which binds separately presented items before making a decision. In addition, some studies documented that females tend to outperform males in perceptual tasks in which subjects must rapidly identify matching items [73]. The negative correlation between the males’ scores and the rGMV of the Medial FC may also support this notion.

There were some similarities between the results of our study and those of the first study which attempted to explore the sex specific neural mechanisms of general intelligence conducted by Haier et al. [38]. Haier’s study used the WAIS to assess general intelligence and observed that the correlation between gray matter volume and intelligence was stronger in the frontal and parietal lobes (BA 8, 9, 39, and 40) for males, whereas a stronger correlation was found in the frontal lobe (BA10) along with Broca’s area for females. It is well known that the Raven’s Matrices test is also a good assessment of general intelligence [2], [39]–[42]. Therefore, although the two studies used different measures of general intelligence, they both yielded results which support the notion that males and females may use different brain structures in different ways to achieve similar levels of general intelligence [39].

In conclusion, this study provided evidence supporting the gender-specific neuroanatomical structure in reasoning performance as measured by the CRT. The results suggest that males rely more on the DLPFC associated with visuospatial ability to achieve a high reasoning score, whereas females rely more on the IFC connected to verbal processing ability and the medial FC associated with information binding ability. No significant differences between genders in the behavioral results of reasoning performance as assessed by CRT were found. These results may indicate that males and females use different brain structures in different ways to achieve similar levels of overall Raven’s reasoning performance. One limitation of this study was that participants were college students who may all share a similar level of reasoning performance. Thus, caution should be taken before generalizing these explanations to a wider population.
